# Effects of broad-leaved grass inhibitors and nitrogen fertilizer on seed production *Elymus nutans* in alpine meadow of the Qinghai-Tibet Plateau

**DOI:** 10.3389/fpls.2025.1470430

**Published:** 2025-02-17

**Authors:** Xin Lu, Juan Qi, Junhu Su, Yanjun Liu, Aolong Zhang

**Affiliations:** ^1^ Grassland Ecosystem Key Laboratory of Ministry of Education, Sino-U.S, Lanzhou, China; ^2^ Grassland Pratacultural College of Gansu Agricultural University, Lanzhou, Gansu, China

**Keywords:** alpine meadow, broad-leaved grass inhibitor, nitrogen fertilizer, *Elymus nutans*, seed yield, seed yield components

## Abstract

**Introduction:**

The alpine meadows of the Tibetan Plateau play a crucial role in the grassland ecosystem. However, due to the rapid growth and strong competitiveness of broad-leaved grasses, the nutritional resources and living space available for Gramineae species are severely restricted in this region. Broad-leaved grasses and noxious weeds have evolved into dominant population, severely limiting grassland production in alpine meadows. A shortage of premium seeds limits grassland ecosystem restoration efforts. *Elymus nutans* is regarded as a pioneer plant for restoring degraded grassland into meadows dominated by grasses, and for developing cultivated grassland in the Tibetan Plateau region, and the demand for native seeds of *E. nutans* is increasing.

**Methods:**

Therefore, this study investigated the effect of combinations of four levels of a broad-leaved grass inhibitor (0, 0.9, 1.5, and 2.1 kg·hm^-2^) crossed with four levels of nitrogen fertilizer (0, 75, 150, and 225 kg·hm^-2^) on seed production of *E. nutans* in Gannan alpine meadow of the Qinghai-Tibet Plateau.

**Results:**

We observed that the grass inhibitor significantly (*p* < 0.05) influenced on fertile tillers (FT), spikelets per fertile tiller (SFT), seeds per spikelet (SS) and panicle length (PL), but not florets per spikelet (FS) (*p* = 0.145). Nitrogen fertilizer significantly influenced on FT, FS, SS, and PL (*p* < 0.001), but not SFT (*p* = 0.068). The interaction of the grass inhibitor and nitrogen fertilizer had no significant effect on any of these seed yield components (*p* > 0.05). Both the grass inhibitor and nitrogen fertilizer significantly influenced all indicators of seed production (*p* < 0.001), increasing their values in a dose-dependent manner. Moreover, their interaction proved significant for all indicators (*p* < 0.001), except for actual seed yield (*p* > 0.05), demonstrating their synergistic effects. The maximum thousand seed weight (4.66 g) and actual seed yield (365 kg·hm^-2^) were observed at the highest doss of 2.1 kg·hm^-2^ of grass inhibitor and 225 kg·hm^-2^ of nitrogen fertilizer, which were 1.85-fold and 2.94-fold of the control, respectively. Furthermore, significantly positive correlations were observed among seed yield and all yield components. Pathway analysis showed that FT made significant direct contributions to the seed yield.

**Discussion:**

This approach (using broad-leaved grass inhibitors and nitrogen fertilizer) effectively reduced competition from broad-leaved grasses and increased the proportion of *E. nutans* in the plant community composition, thus alleviating the shortage of *E. nutans* seeds for grassland ecological restoration.

## 1 Introduction

The Qinghai-Tibet Plateau stands as the largest alpine grassland distribution area globally (Dong and Sherman, 2015). Encompassing over 60% of the entire Tibetan Plateau area (Zhang et al., 2014; Jesús, 2019). Alpine meadows, the largest alpine ecosystem on the Tibet Plateau, this ecosystem serves crucial ecological functions, such as biodiversity maintenance, acting as an ecological barrier, and supporting livestock production on a global scale (Dong et al., 2020). However, a myriad of natural and anthropogenic factors have led to varying degrees of degradation across most grasslands (Pan et al., 2023). Overgrazing is considered the primary cause of degradation in alpine meadows. Prolonged overgrazing resulted in excessive overeating of Gramineae species favored by cattle and sheep, leading to a sharp decline in the number of Gramineae species and a significant reduction in the Gramineae functional group (Li et al., 2015a; Liu et al., 2014; Li et al., 2015b). Original dominant and key species were gradually declining, while toxic, unpalatable, and low-nutrition plant species proliferated, potentially covering up to 80% of the area (Li et al., 2019). The dominance of Gramineae and Sedge communities struggles to regenerate quickly, while broad-leaved grasses and noxious weeds thrive under similar nutrient conditions, thus preventing normal growth and reproduction in *E. nutans* (Li et al., 2020). Over time, broad-leaved grasses and noxious weeds have evolved into dominant population, severely limiting grassland production in alpine regions. Presently, the proliferation of broad-leaved grasses poses a significant threat to the ecological integrity of alpine meadow and the sustainability of animal husbandry.

As grassland ecological restoration efforts continue, alongside the large-scale development of animal husbandry and the dairy industry, as well as the implementation of ecological management projects such as “Grain to Green” and “Returning Farmland to Grassland” (Dai et al., 2023), there is a pressing need for the restoration of vast areas of degraded alpine grassland through revegetation (Gao et al., 2019). This has led to a rapid increase in demand for high-quality grass seeds. Native grass seeds that are compatible with the environmental conditions of the target restoration regions are especially in demand (Du, 2021). According to available data and statistics, the national ecological construction plan predicts that over the next five years, more than 70,000 tons of grass seeds per year will be required for the improvement of degraded grasslands and ecological environment management. This highlights a significant gap between the supply and demand of grass seeds (Nan et al., 2022; Yang and Wang, 2023). Numerous ecological restoration experiments have underscored the pivotal role of high-quality grass seeds supply in restoration efforts. However, relatively few studies have delved into the dependence of seed viability on parent plant conditions (Elzenga et al., 2019). Previous research indicated that certain exogenously introduced species serve as primary candidates for ecological restoration. Nonetheless, while these species exhibit robust growth in the initial 2~3 years post-seeding, degradation of the reseeded grassland becomes apparent from year 4 onwards. This degradation could be attributed to the poor ecological adaptability of the seeds to local climate and environmental changes (De, 2014; Yang and Wang, 2023). Some studies have explored cultivated seed production methods to generate native seeds in various countries. However, cultivated native seed production may results in the loss of adaptations to specific natural habitats or reduced genetic diversity (Espeland et al., 2017; Simone et al., 2020). Currently, seeds used in ecological restoration were predominantly sourced from natural populations in many countries (Simone et al., 2020). While wild harvesting provides seeds reflective of genetic diversity of native species and resilience, it often falls short of meeting the demands for large-scale restoration. The scarcity of high-quality native grass seeds, particularly from the Gramineae family, has emerged as a major constrain in restoration ecology, hindering the attainment of desirable restoration outcomes (Du, 2021; Dai et al., 2023). Consequently, finding solutions to expedite the production of high-quality Gramineae native seeds and improving grassland stability remains an urgent issue in current restoration endeavors.

The primary native species of Gramineae grasses in the alpine pasture area of Gannan Tibetan Autonomous Prefecture, *Elymus nutans*, is often regarded as a pioneer plant for restoring degraded grassland into meadows dominated by grasses, as well as for developing cultivated grassland in the Tibetan Plateau region (Zhang et al., 2023). With the accelerated restoration of degraded grasslands and the expansion of cultivated grassland areas, the demand for native seeds of *E. nutans* is increasing. Given the degradation status of alpine meadows and the severe shortage of suitable native seeds of *E. nutans* for grassland ecological restoration, a viable intervention strategy involves inhibiting the growth of broad-leaved grasses in degraded grasslands, which can reduce the competition among broad-leaved grasses and increase the proportion of *E. nutans* in the structural composition of plant communities, thereby addressing the scarcity of *E. nutans* seeds for grassland ecological restoration.

Studies have also demonstrated that the nitrogen can be absorbed and utilized by extremely nitrogen-sensitive plants, such as Gramineae grasses over a short period, thereby enhancing the ability of Gramineae grasses to occupy ecological niches and improving seed yield (Cao et al., 2015). Consequently, selectively regulating forage growth using highly effective inhibitors of broad-leaved grasses can increase seed yield of *E. nutans* in grassland communities. Coupling this approach with nitrogen fertilizer can further adjust the proportion of dominant grasses in the soil seed bank, promoting desirable restoration outcomes, biodiversity, and ecosystem functions. Therefore, we devised a two-level split-plot experiment to analyze the relationships between seed yield characteristics of *E. nutans* in response to various combinations of a broad-leaved grass inhibitor and nitrogen fertilizer levels. Subsequently, we aimed to determine the optimal combination between broad-leaved inhibitor and nitrogen fertilizer level for improving seed yield of *E. nutans* in the alpine meadow of the Qinghai-Tibet Plateau region.

## 2 Materials and methods

### 2.1 Study site

**Figure 1:**
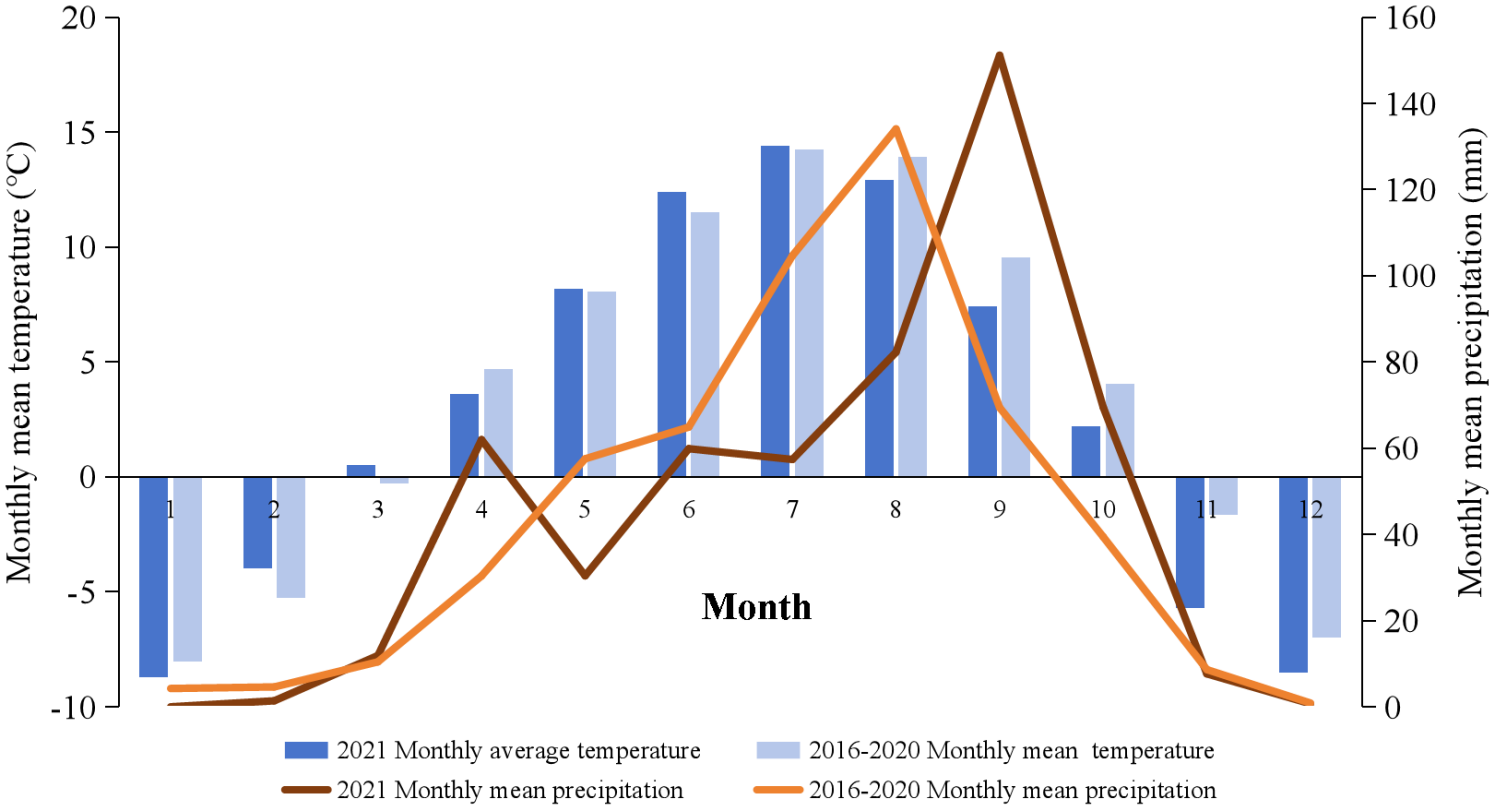
The monthly mean temperature and precipitation in the study site from 2016 to 2020, along with 2021.

The study site is situated in Sangke grassland, within Xiahe County, Gannan Tibetan Autonomous Prefecture, in Gansu Province, China (102°21’-102°29’ E, 34°59’-35°09’ N), respecting a typical alpine meadow. The average altitude of the location is 3,047 m. The monthly mean precipitation and temperature distributions are shown in **Figure 1**. The mean monthly temperature stands at 3.4°C, with an average annual precipitation approximately 449 mm, mainly occurring during the plant growing season (June to September). Annual sunshine hours total 2,425, and the frost-free period extends over 58 days annually. The climate type is categorized as a cold and humid plateau continental climate.

The field experiment was conducted in the Sangke alpine winter-early spring pasture, which banned grazing during summer and autumn seasons, from early May to early November. The grassland is classified as medium degraded grassland, with dominant species including *E. nutans* and *Kobresia humilis*. The experiment site exhibited uniform density. The soil is characterized by alpine meadow soil with zonal soil properties, as detailed in **Table 1**, which also lists the chemical properties of soil.

**Table 1:** Chemical characteristics of soil in the study site.

Organic matter (g·kg^-1^)	Total nitrogen (g·kg^-1^)	Total phosphorus (g·kg^-1^)	Total potassium (g·kg^-1^)	Available nitrogen (mg·kg^-1^)	Available phosphorus (mg·kg^-1^)	Available potassium (mg·kg^-1^)
73.12	3.96	0.49	10.62	270	26	207

### 2.2 Experimental design

This experiment utilized a two-factor split-plot experimental design, involving the application of a broad-leaved grass inhibitor in combination with nitrogen fertilizer. The broad-leaved grass inhibitor (Y) was applied at four levels: 0 (Y0), 0.9 (Y0.9), 1.5 (Y1.5) and 2.1 (Y2.1) kg·hm^-2^, respectively. Nitrogen fertilizer, in the form of urea (N ≥ 46.0%), was also utilized at four levels: 0 (N0), 75 (N75), 150 (N150) and 225 (N225) kg·hm^-2^, respectively. Consequently, there were a total of 16 treatments, with each treatment compromising three subplots measuring 15 m × 15 m, and with 1.5 m distance between any two plots.

The experiment was conducted from June to October of 2021, in a selected area characterized by uniform vegetation and relatively flat terrain. The broad-leaved grass inhibitor used in this study was a mixture of MCPA and Fluroxypyr herbicides (Beijing Linhai Plant Protection Technology Co., China), with a total active ingredient content of 42%, consisting of 33.5% MCPA and 8.5% Fluroxypyr. During the flourishing period of broad-leaved grasses in early June, the broad-leaved grass inhibitor (0 g, 20.25 g, 33.75 g, 47.25 g) was thoroughly mixed with 6.7 L water and sprayed onto each plot using a backpack hand sprayer. To ensure complete dissolution of urea in the soil, nitrogen fertilizer was applied to the soil surface. Rainy days were chosen to spray nitrogen fertilizer, allowing for its incorporation into the surface soil during the tillering stage of *E. nutans* in early July.

### 2.3 Seed yield components

During the peak flowering stage, the number of reproductive tillers was counted by harvesting three quadrats, each measuring 1.0 m by 1.0 m from separate areas near the center of each subplot. Ten fertile tillers and spikelets were selected randomly to determine the number of spikelets per fertile tiller, as well as florets per spikelet and seeds per spikelet, at the milky ripening stage. Additionally, panicle length was measured by randomly selecting ten plants in each subplot and measuring the distance from the node (where the first panicle branch starts) to the tip of the panicle.

### 2.4 Seed yield

In this analysis, the weight of hundred seeds was determined in triplicates, and converted to the thousand seed weight. The potential seed yield and performance seed yield were calculated as follows (Han et al., 1996; Song, 2022):


(1) $$\text{Potential seed yield }(\text{kg}\cdot {\text{hm}}^{-2})={\text{fertile tillers per m}}^{-2}\times \text{spikelet per fertile tiller}\times \text{florets per spikelet}\times \text{average seed weight}\times 10$$

(2) $$\text{Performance seed yield }(\text{kg}\cdot {\text{hm}}^{-2})={\text{fertile tillers per m}}^{-2}\times \text{spikelet per fertile tiller}\times \text{seed number per spikelet}\times \text{average seed weight}\times 10$$

The actual seed yield of *E. nutans* was determined at maturity of the plants in mid-September, 2021. Quadrats measuring 1 m × 1 m were selected in each subplot, excluding the marginal areas. These quadrats were collected and then transported to the laboratory for air-drying, cleaning, and subsequent weighing, with each process repeated three times. The actual seed yield of *E. nutans* was determined.

### 2.5 Statistical analyses

This experiment followed a two-factor split-plot experimental design with triplicates for each treatment. Therefore, a two-way ANOVA model was employed to analyze the effects of the broad-leaved grass inhibitor and nitrogen fertilizer (as factors in the model), as well as their interaction, on the seed yield characteristics of *E. nutans*. In instance where the interaction between the two factors was not statistically significant (*p* > 0.05), the means for each level of the grass inhibitor across all four nitrogen fertilizer levels, or for each level of nitrogen fertilizer across all four grass inhibitor levels, along with the corresponding standard error of means (SEM, n = 12) were presented. When the interaction between the two factors was significant (*p* < 0.05), the means for each of the 16 combinations were presented with SEM (n = 3). The multiple comparison of the means was performed using Duncan’s method. Additionally, correlation and pathway analyses were carried out to explore the relationships between seed yield and seed yield components. Statistical significance was declared when *p*-values ≤ 0.05. Statistical analysis was performed using SPSS software (version 26, IBM Corporation, Armonk, NY, USA). Figures were generated using Origin Pro software (version 2021, Origin Lab, Corporation, Northampton, MA, USA).

## 3 Results

### 3.1 Seed yield components

Five indices for seed yield components, namely the number of fertile tillers per m^2^ (FT), the number of spikelets per fertile tiller (SFT), the number of florets per spikelet (FS), the number of seeds per spikelet (SS), and the panicle length (cm, PL), are presented in **Table 2**. The grass inhibitor had significant effects on FT, SFT, SS, and PL (*p* < 0.05), but not on FS (*p* = 0.145). Nitrogen fertilizer significantly influenced on FT, FS, SS, and PL (*p* < 0.001), but not SFT (*p* = 0.068). The interaction of the grass inhibitor and nitrogen fertilizer had no significant effect on any of these seed yield components (*p* > 0.05).

**Table 2:** Effects of the broad-leaved grass inhibitor and nitrogen fertilizer on seed yield components of *E. nutans*.

	Treatments	Fertile tiller/m^2^ (FT)	Spikelet per fertile tiller (SFT)	Florets per spikelet (FS)	Seeds per spikelet (SS)	Panicle length (cm) (PL)
Y	N0	258a	28.64a	3.08a	2.02a	10.85a
	N75	314b	29.66ab	3.43b	2.41b	12.40b
	N150	376c	31.22ab	3.50b	2.55b	12.62b
	N225	419d	32.06b	3.73c	2.92c	13.79c
N	Y0	282A	27.49A	3.29A	2.23A	11.60A
	Y0.9	335B	30.52B	3.48AB	2.32AB	12.42A
	Y1.5	352B	31.28B	3.52B	2.56B	12.73A
	Y2.1	400C	32.29B	3.44AB	2.78B	12.91B
SEM		8.67	0.950	0.0716	0.1279	0.248
Significance (*p* value)						
Y		<0.001	0.008	0.145	0.021	0.004
N		<0.001	0.068	<0.001	<0.001	<0.001
Y×N		0.220	0.874	0.342	0.967	0.451

Lowercase letters within a column indicate significant differences between nitrogen fertilizer levels (*p* < 0.05), and uppercase letters represent significant differences between grass inhibitor levels (*p* < 0.05).

As shown in **Table 2**, the grass inhibitor consistently increased the numbers of these five seed yield components in a dose-dependent fashion, with the greatest values observed at the highest dose of the inhibitor, except for the number of FS, which reached its peak at inhibitor dose of 1.5 kg·hm^-2^. In addition, compared to Y0, the inhibitor significantly increased the number of SFT at dose of 0.9 kg·hm^-2^, and SS at dose of 1.5 kg·hm^-2^. The influences of nitrogen fertilizer on the five seed yield components exhibited a similar trend to that of the grass inhibitor. Specifically, the values of each component consistently increased with the nitrogen level (*p* < 0.05) and reached their peaks at the highest level of nitrogen fertilizer. Compared to N0, FT, SFT, FS, SS, and PL increased by 62%, 12%, 21%, 45%, and 27%, respectively, at a nitrogen fertilizer level of 225 kg·hm^-2^.

### 3.2 Seed yield

The seed production indicators, namely thousand seed weight, potential seed yield, seed performance yield, and actual seed yield of *E. nutans* in response to each level of the grass inhibitor and nitrogen fertilizer, are shown in **Table 3**. Both the grass inhibitor and nitrogen fertilizer significantly influenced all indicators of seed production (*p* < 0.001), increasing their values in a dose-dependent manner. Moreover, the interaction between the grass inhibitor and nitrogen fertilizer proved significant for all indicators (*p* < 0.001), except for actual seed yield (*p* > 0.05), demonstrating their synergistic effects. Therefore, the means of each of the 16 combinations of the grass inhibitor and nitrogen fertilizer are presented in the table. As shown in **Table 3**, the thousand seed weight reached its maximum values in combinations of Y1.5+N225, Y2.1+N150, and Y2.1+N225, while the potential seed yield, seed performance yield, and actual seed yield peaked in the combination of Y2.1+N225. Compared to the combination of Y0+N0, their peak values increased by 0.85-fold for the thousand seed weight, 8.56-fold for the potential seed yield, 10.48-fold for the seed performance yield, and 2.94-fold for the actual seed yield, respectively.

**Table 3:** Effects of the broad-leaved grass inhibitor and nitrogen fertilizer on the indicators of seed production of *E. nutans*.

Treatments		Thousand seed weight (g)	Potential seed yield (kg·hm^-2^)	Seed performance yield (kg·hm^-2^)	Actual seed yield (kg·hm^-2^)
Y0	N0	2.52a	372a	264a	124a
	N75	3.28b	704b	484ab	132ab
	N150	3.78cd	1228e	825cd	185cd
	N225	4.02cde	1459f	994cde	206cde
Y0.9	N0	3.36b	831bc	441ab	165bc
	N75	3.72c	1235e	830cd	208de
	N150	3.93cde	1627fh	1065def	228ef
	N225	4.12def	1858i	1381f	209de
Y1.5	N0	3.94cde	983cd	694bc	183cd
	N75	3.95cde	1459fg	962cde	207de
	N150	4.19ef	1811i	1234ef	235ef
	N225	4.50g	2399k	2000g	267fgh
Y2.1	N0	3.99cde	1137de	818cd	255fg
	N75	4.17ef	1605fgh	1213ef	286gh
	N150	4.39fg	2085j	1845g	306h
	N225	4.66g	3186l	2767h	365i
SEM		0.102	54.4	104.2	13.0
Significance (*p* value)					
Y		<0.001	<0.001	<0.001	<0.001
N		<0.001	<0.001	<0.001	<0.001
Y×N		0.001	<0.001	<0.001	0.121

Lowercase letters within a column indicate significant differences between the means (*p* < 0.05).

**Figure 2:**
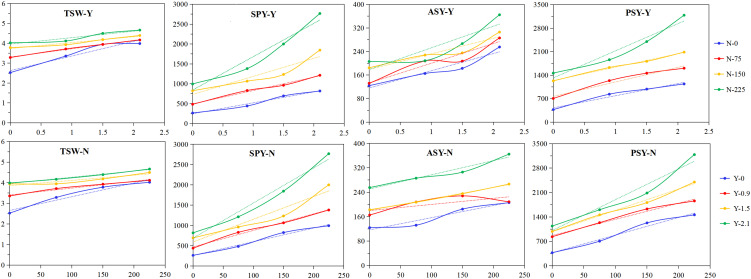
Effects of the broad-leaved grass inhibitor and nitrogen fertilizer on the seed production indicators of *E. nutans.* TSW, thousand seed weight (g); ASY, actual seed yield (kg·hm^-2^); SPY, seed performance yield (kg·hm^-2^); PSY, potential seed yield (kg·hm^-2^). Y, the broad-leaved grass inhibitor; N, nitrogen fertilizer. The top four figures show the means for each grass inhibitor level across four nitrogen fertilizer levels, and the bottom four figures exhibit the means for each nitrogen level across four the grass inhibitor levels.

To demonstrate the relationships between the seed production indicators and the grass inhibitor or nitrogen fertilizer, the means for each level of the grass inhibitor across all four nitrogen fertilizer levels, or for each level of nitrogen fertilizer across all four grass inhibitor levels, are depicted in **Figure 2**. As illustrated in the figure, all seed production indicators exhibited increases with the grass inhibitor level, but the extent of the increments varied depending on the nitrogen fertilizer level, and vice versa. For instance, the increase in thousand seed weight from 0 to 2.1 kg·hm^-2^ of the grass inhibitor was 158%, 127%, 116%, and 116%, respectively, for nitrogen fertilizer application of 0, 75, 150, and 225 kg·hm^-2^. Similarly, the increase in thousand seed weight from 0 to 225 kg·hm^-2^ of nitrogen fertilizer was 160%, 123%, 114%, and 117%, respectively, for the grass inhibitor level of 0, 0.9, 1.5, and 2.1 kg·hm^-2^. As for actual seed yield, the increase from 0 to 2.1 kg·hm^-2^ of the grass inhibitor was 206%, 170%, 165%, and 177%, respectively, for nitrogen fertilizer application of 0, 75, 150, and 225 kg·hm^-2^; and the increase from 0 to 2.1 kg·hm^-2^ of the grass inhibitor was 166%, 127%, 146%, and 1436%, respectively, for nitrogen fertilizer application of 0, 75, 150, and 225 kg·hm^-2^.

### 3.3 Relationships between seed yield components and seed yield indicators

The correlations between the seed yield components (FT, SFT, FS, SS, and PL) and thousand seed weight and actual seed yield were summarized in **Table 4**. All components exhibited significant correlations with the seed weight and yield (*p* < 0.01), with correlation coefficients ranging from 0.537 to 0.848 for thousand seed weight. The rank of the coefficients was as follows: FT, PL, SS, SFT, and FS.

**Table 4:** Correlations between seed yield components and seed weight and yield of *E. nutans*.

	FT	SFT	FS	SS	PL	TSW	Actual seed yield
FT	1.000	0.543**	0.628**	0.630**	0.723**	0.848**	0.822**
SFT		1.000	0.266 ns	0.344*	0.515**	0.561**	0.513**
FS			1.000	0.466**	0.595**	0.537**	0.449**
SS				1.000	0.492**	0.592**	0.498**
PL					1.000	0.662**	0.588**
TSW						1.000	0.776**
Actual seed yield							1.000

FT, Fertile tillers; SFT, Spikelet per fertile tiller; FS, Florets per spikelet; SS, Seeds per spikelet; PL, Panicle length; TSW, Thousand seed weight; *p < 0.05; **p < 0.01 level; ns, No significance (*p* > 0.05).

**Figure 3:**
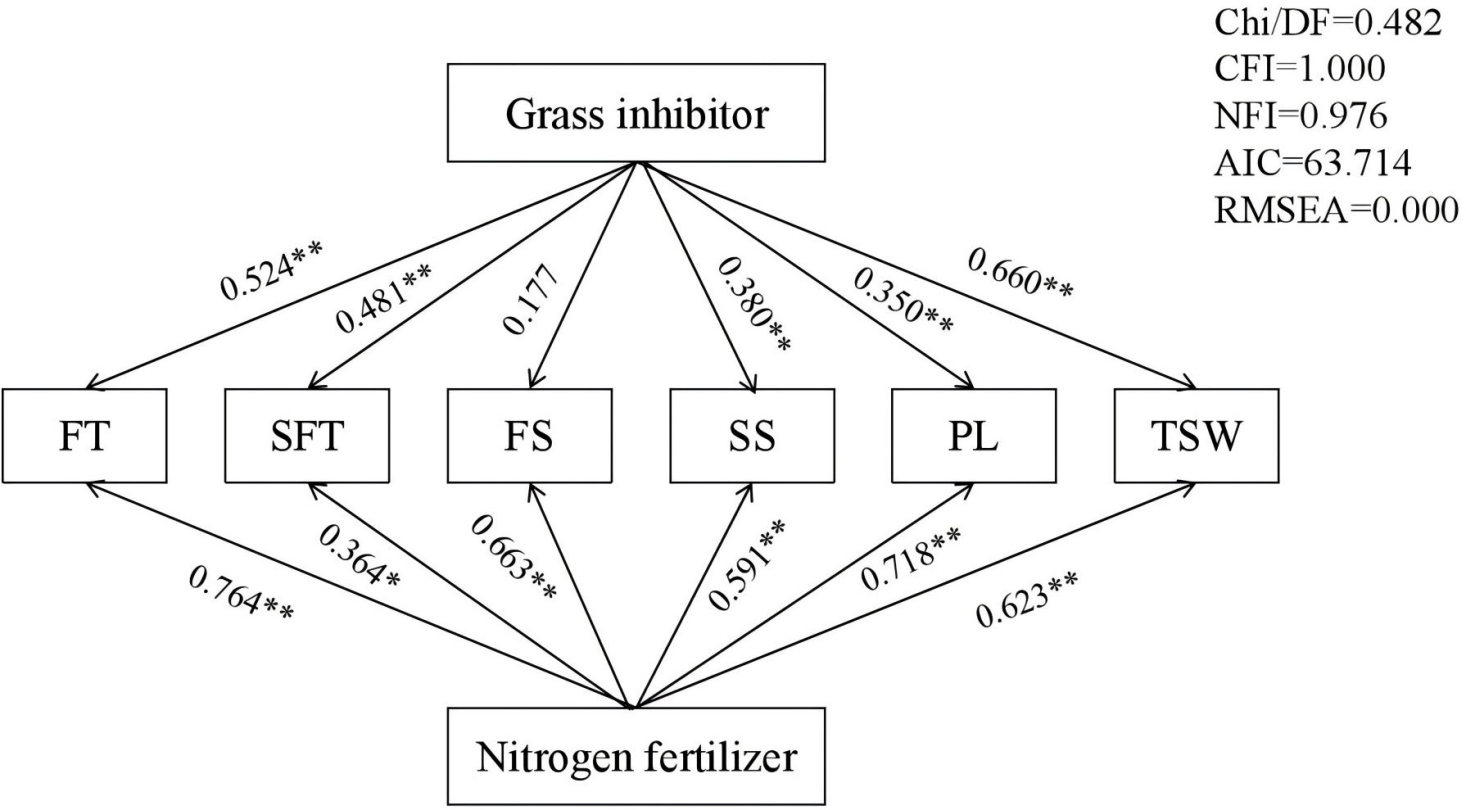
Pathway analysis of the broad-leaved grass inhibitor and nitrogen fertilizer on seed yield components and seed weight of *E. nutans.* The numbers displayed on the lines represent pathway coefficients. *p < 0.05; **p < 0.01.

Pathway analysis was conducted to explore the contributions of the grass inhibitor and nitrogen fertilizer to the seed yield components (**Figure 3**). The analysis revealed that the grass inhibitor exerted a direct positive influence on the seed yield components, expect for FS. Conversely, nitrogen fertilizer exhibited a direct positive influence on all seed yield components, with the highest correlation coefficient of 0.764 observed for FT. The multiple stepwise regression analysis demonstrated that FT significantly (*p* < 0.01) contributed to seed yield with direct pathway coefficient of 0.668 (**Table 5**). Furthermore, the path analysis utilized six seed yield components to determine direct, indirect, and total path coefficients for each component. Among these, FT displayed the highest total path correlation coefficient (0.823), while, FS exhibited the lowest total path correlation coefficient (0.018). This low total path correlation coefficient for FS stemmed from its minimum path coefficient and low correlations.

**Table 5:** Pathway analysis for direct and indirect effects of seed yield components of *E. nutans*.

	FT	SFT	FS	SS	PL	TSW
Direct pathway coefficients	0.668	0.048	-0.096	-0.048	-0.023	0.279
Indirect pathway coefficients:						
SFT	0.026	–	–	–	–	–
FS	-0.060	-0.026	–	–	–	–
SS	-0.030	-0.017	-0.022	–	–	–
PL	-0.017	-0.012	-0.014	-0.011	–	–
TSW	0.237	0.157	0.150	0.165	0.185	–
Total path coefficients	0.823	0.151	0.018	0.106	0.162	0.279

## 4 Discussion

### 4.1 Effects of grass inhibitor and nitrogen fertilizer on seed yield and yield components in *E. nutans*

In the alpine meadows of the Tibetan Plateau, a significant challenge often encountered is vigorous growth of broad-leaved grasses. These grasses serve as a critical limiting factor in the cultivation of high-quality grasses in alpine meadows, such as *E. nutans*. Consequently, controlling the growth of broad-leaved grasses becomes essential for enhancing the seed yield of premium pasture grasses (Feng et al., 2016). By targeting specific plants, grass inhibitors directly or indirectly influence the growth of others, thereby enhancing their nutrient uptake and soil nutrient metabolism (Viktoria and Cara, 2014; Rachelle et al., 2023). Forage seed yield encompasses three types: potential seed yield, also known as theoretical seed yield, performance seed yield, and actual seed yield. The measurable factors contributing to seed yield are referred to as seed yield components, each of which is interconnected with seed yield. Investigating seed yield components to identify factors limiting seed yield increase provides a theoretical foundation for enhancing seed yield (Zhao, 2003). In the case of Gramineous forages, seed yield components such as fertile tillers, spikelet per fertile tiller, florets per spikelet, seeds per spikelet, among others, directly contribute to seed yield (Zerang, 2019; Ou et al., 2021). Several studies have indicated that enhancing these traits was an effective approach to boosting seed yield (Abel et al., 2017).

This study found that the grass inhibitor exerted potent inhibitory effects on broad-leaved grasses. Following the application of the broad-leaved grass inhibitor, it was observed that the seed yield and associated seed yield components of *E. nutans* increased, and the highest seed yield was attained at the highest dose of 2.1 kg·hm^-2^. These findings suggest that the inhibitor had a positive impact on enhancing the seed yield of *E. nutans* within a specific concentration range. Presumably, the application of this inhibitor through spraying effectively suppressed the growth and development of broad-leaved grasses, thereby reallocating more available resources to Gramineae plants, such as water, soil nutrients, light, and space. Consequently, the proportion of high-quality grasses within the community significantly rose, consequently impacting the seed yield and spike traits of Gramineae (Liu et al., 2022). A previous study reported that the inhibitors may regulate the growth of broad-leaved weeds while limiting the formation of new tiller buds. This reduction in tiller bud formation decreases the competitiveness of broad-leaved grasses, thereby, mitigating competition for nutrients and space between new and old branches within the Gramineae community; consequently, this situation results in an increase in the number of reproductive branches and a higher seed yield (Liu and You, 2010).

Investigations have revealed various limitations to seed production within plant communities. In alpine meadows, apart from the impact of broad-leaved grasses, soil infertility emerges as a significant constraint to the increased seed yield of *E. nutans*. Several studies have shown that fertilization can enhance the proportion of each seed yield component, with nitrogen fertilization identified as a key influence on seed yield (Catuchi et al., 2017). The results of this study underscored the substantial impact of nitrogen fertilizer application on both seed yield and its components of *E. nutans*, following a dose-dependent pattern, a trend consistently observed in other studies (Qiao et al., 2006). Furthermore, the results in the present study indicated a significant increase in Gramineae seed yield due to nitrogen fertilizer application, primarily attributed to the heightened numbers of FT and SS (Wang, 2018). In this experiment, compared to the control (no nitrogen application), the number of fertile tillers (FT) significantly increased with the increasing nitrogen application, with 62.4% increase under the high nitrogen treatment. Study has also shown that, within a certain range, nitrogen fertilization could significantly increase the number of reproductive branches per unit area of forage grasses, thereby enhancing seed yield (Li et al., 2023). Chen et al. (2005) found that when planting *Festuca arundinacea* in Haidian District, Beijing, the optimal nitrogen application rate was 150-180 kg·hm^-2^, and the number of reproductive branches significantly increased with the increasing nitrogen application rate. Notably, numerous studies highlight the pivotal role of spike traits in overall yield (Sartie et al., 2018; Zhong et al., 2020), with spike type identified as a decisive factor influencing seed yield (Nouraein, 2019). Moreover, nitrogen fertilizer application, regardless of its level, exerted a notable influence on the thousand seed weight of *E. nutans*, a variable that demonstrated a significant increase with higher nitrogen application amounts in this study, a trend commonly reported in other studies (Xie et al., 2010). Previous research has underscored nitrogen fertilizer application as a primary determinant of seed yield. Within a specific range, increased nitrogen fertilizer application has been associated with higher seed yield, irrespective of the presence of fertilizer types (Canto et al., 2020; Yuan et al., 2022). In the present study, nitrogen fertilizer application substantially elevated all seed yield indicators. The yield of forage seeds is heavily contingent upon climatic conditions and grassland management practices. A growing body of literature suggests that nitrogen fertilizer, as the primary fertilizer used on the Tibetan Plateau, can be readily absorbed and utilized by highly nitrogen-sensitive plants such as Gramineae within a short time frame. This rapid absorption enhances the ability of Gramineae to occupy ecological niches, thereby positively contributing to seed yield and its components (Cao et al., 2015).

Surprisingly, a substantial disparity between the potential seed yield and the actual final seed yield of *E. nutans* was observed in the present study. Theoretically, all *E. nutans* seed yields should be high; however, actual seed yields in this study amounted to only about 10-20% of potential seed yields, or even lower. Moreover, we observed that when nitrogen fertilizer exceeded 150 kg·hm^-2^, the discrepancy in actual seed yield between medium and high nitrogen fertilizer treatments was not significantly different. Although the number of fertile tillers was higher in 225 kg·hm^-2^ nitrogen fertilizer than in those with 150 kg·hm^-2^, the actual seed yield did not exhibit a significant difference between two nitrogen fertilizer levels. This phenomenon may be attributed, in part, to the fact that after the application of nitrogen fertilizer, *E. nutans* prioritized vegetative growth over reproductive development, affecting the allocation of energy for nutrient growth and reproductive processes (Scotton, 2018; Xu et al., 2023). Furthermore, July-September rainfall, a crucial component of annual rainfall in this study region, severely limited pollination and seed formation of *E. nutans* (Stephenson, 1981; Dupont et al., 2018). Additionally, the seed shattering (seed abscission) increased during harvesting as seeds approached maturity (Tanentzap and Monks, 2018), resulting in lower actual seed yields.

### 4.2 Coupled effects of inhibitors and nitrogen on seed yield in *Elymus nutans*

The present study demonstrated that the interaction between the broad-leaved grass inhibitor and nitrogen application had a highly significant effect on the seed yield of *E. nutans*. However, the impact on seed composition factors was not significant. It can be concluded that for yield components such as FT, SS, and SFT, the individual effects of the inhibitor and nitrogen application were more significant, while their interaction effect was not significantly manifested. This finding suggested that future research should investigate the specific mechanisms affecting different yield components, particularly how to balance nitrogen fertilizer application and inhibitor concentration to optimize all yield components. Futhermore, the combined effect of measures (Y×N) was more significant in increasing seed yield than the individual application of either the inhibitor or nitrogen alone. The current study revealed a synergistic effect between the broad-leaved grass inhibitor and nitrogen fertilizer on seed yield. The inhibitor and nitrogen application significantly affected the thousand-seed weight and seed yield. The highest increases in thousand-seed weight and seed yield were observed with inhibitor concentration of 2.1 kg·hm^2^ and nitrogen application rate of 225 kg·hm^2^, resulting in 2.94-fold increase in seed yield and 0.79-fold increase in thousand-seed weight.

### 4.3 Relationships between seed yield and constituent factors

Correlation analysis can elucidate the relationship between seed yield and its constituent factors, identify constraints on increasing seed yield, and unveil novel pathways for enhancing seed yield production. In the context of *E. nutans*, the rank order of the effects of its six yield components on seed yield was found to be FT > TSW > PL > SFT > SS > FS. This hierarchy indicated that FT, influenced significantly by the broad-leaved grass inhibitor and nitrogen fertilizer, exerted strong direct effects on seed yield, corroborating early findings (Ou et al., 2021).

Pathway analysis revealed direct positive influences of the grass inhibitor and nitrogen fertilizer on seed yield components, with the maximum correlation coefficients of 0.524 and 0.764 observed for FT (**Figure 3**). Multiple stepwise regression analysis further demonstrated that FT significantly contributed directly to seed yield, with a direct pathway coefficient of 0.668 and the highest total path coefficient correlation of 0.823. Conversely, FS exhibited a low direct pathway coefficient and a low correlation coefficient. The application of nitrogen fertilizer during the tillering stage was found to enhance the production of fertile tillers, fostering spikelets and seed developments, and facilitating the accumulation of photosynthesis products, thereby improving seed yield. Previous studies have shown the complementary effects among the seed yield components in maintaining overall seed yield of *E. nutans* (Ou et al., 2021). During periods of adverse weather conditions, the complementary effects of these seed yield components are instrumental in balancing the seed yield production. To obtain high seed yields, each yield component must be cultivated in harmony (Liu et al., 2022). Therefore, implementing measures to balance yield components in natural grassland managements represents the most direct and effective strategy for increasing seed yield.

### 4.4 A comprehensive assessment

Due to the inadequate conditions for human management in the natural grasslands of the alpine region, broad-leaved grasses exhibit high competitiveness, dominating much of the community’s living space and resulting in low yields of high-quality native grasses. The primary objective of interventional restoration measures, such as fertilization, is to increase native seed production within the targeted area. This study achieved significant results by inhibiting the growth points of broad-leaved grasses during their early growth stage. Among various functional groups, Gramineous forages such as *E. nutans* are highly sensitive to nitrogen and can efficiently absorb and utilize nitrogen fertilizer to accelerate growth in a short period of time. During the process of inhibiting the growth of mixed weeds, the application of nitrogen fertilizer enables *E. nutans* to rapidly enhance their competitive ability, particularly in terms of nutrient and space acquisition, allowing them to quickly occupy the upper space. This not only effectively suppressed the growth and reproductive success of broad-leaved grasses but also promoted the growth and development of Gramineous species, significantly increasing the seed yield of high-quality Gramineous grasses. In this experiment, compared to the control (Y0N0), seed yield under the Y2.1+N150 and Y2.1+N225 treatments increased by 146.77% and 195.35%, respectively. Additionally, to prevent lodging of *E. nutans* and considering cost-effectiveness, it was observed that the optimal nitrogen application rate for native grass seed production of *E. nutans* in alpine meadows of the Tibetan Plateau is between 150 and 225 kg·hm^-2^. When combined with 2.1 kg·hm^-2^ of broad-leaved grass inhibitor, seed yield can be increased by more than two times. This approach (broad-leaved grass inhibitors and nitrogen fertilizer) effectively reduced the competition among broad-leaved grasses and increased the proportion of *E. nutans* in the structural composition of plant communities, thereby addressing the scarcity of *E. nutans* seeds for grassland ecological restoration.

From an economic perspective, under the high concentration inhibitor and high nitrogen application treatment, the seed yield of *E. nutans* increased by 194.35%, rising from 124 kg·hm^-2^ to 365 kg·hm^-2^. Based on the local seed market price of 23 yuan per kilogram for *E. nutans*, the income per hectare was 5543 yuan. After subtracting the input costs for the first year (inhibitor + nitrogen) of about 900 yuan per hectare, the net economic benefit per hectare was 4643 yuan. By promoting the mass propagation of native grass species, it helps address one of the major bottlenecks in ecological restoration in alpine regions—namely, the issue of sourcing high-quality supplemental grass seeds. This, in turn, contributes to the promotion of local ecological restoration efforts.

Although this study achieved significant results with the combined use of broad-leaved grass inhibitors and nitrogen fertilizer, its limitations cannot be ignored. Firstly, the study was conducted only in the alpine meadow ecosystem of the Tibetan Plateau, and the grassland restoration methods used may be influenced by the local climate, soil conditions, and vegetation types. Therefore, whether this method can be applied to other ecological regions, especially those with different climate and soil conditions, still requires further validation. Future studies should involve broader research, particularly testing in different ecological zones, to assess the universality of this approach and determine whether similar results can be achieved in other regions. Furthermore, unmanned aerial vehicles (UAV) are widely used to obtain high-temporal and high-spatial resolution remote sensing images of crops, enabling a possible sensor performance comparison. In our study, we primarily focused on the combined effects of the inhibitor and nitrogen fertilization on the seed yield of *E. nutans*. Given that multiple factors, such as climate conditions, may have a complex and interactive impact on seed yield, it is recommended to incorporate machine learning models in future research to more comprehensively analyze the interactions of these factors. Machine learning models, such as random forest, neural networks, linear regression, and gradient boosting trees, are capable of effectively handling multidimensional data and offer powerful potential tools for predicting seed yield (Guo et al., 2023; Klompenburg et al., 2020). By integrating machine learning models, we can apply them to our research to gain a deeper understanding of the impact of various factors on seed yield.

## 5 Conclusions

Both the broad-leaved grass inhibitor and nitrogen fertilizer proved effective in enhancing the seed yield components of *E. nutans* in alpine meadows, exhibiting a dose-dependent response. Synergistic effects between the grass inhibitor and nitrogen fertilizer were evident in the seed productivity indicators, with maximum seed weight and yield achieved in a combination of 2.1 kg·hm^-2^ of the grass inhibitor and 225 kg·hm^-2^ of nitrogen fertilizer. Consequently, seed yield increased by 2.94-fold compared to the control. However, the efficacy per unit of the grass inhibitor and nitrogen fertilizer on seed yield was compromised, warranting further economic evaluation. Future studies will necessitate long-term research to validate these findings and to elucidate the regulatory mechanisms of the inhibitors and nitrogen fertilizer.

## Data Availability

The original contributions presented in the study are included in the article/supplementary material. Further inquiries can be directed to the corresponding author.
